# RF-CNN-F: random forest with convolutional neural network features for coronary artery disease diagnosis based on cardiac magnetic resonance

**DOI:** 10.1038/s41598-022-15374-5

**Published:** 2022-07-01

**Authors:** Fahime Khozeimeh, Danial Sharifrazi, Navid Hoseini Izadi, Javad Hassannataj Joloudari, Afshin Shoeibi, Roohallah Alizadehsani, Mehrzad Tartibi, Sadiq Hussain, Zahra Alizadeh Sani, Marjane Khodatars, Delaram Sadeghi, Abbas Khosravi, Saeid Nahavandi, Ru-San Tan, U. Rajendra Acharya, Sheikh Mohammed Shariful Islam

**Affiliations:** 1 Institute for Intelligent Systems Research and Innovation (IISRI), Deakin University, Geelong, Australia; 2 Department of Computer Engineering, School of Technical and Engineering, Shiraz Branch, Islamic Azad University, Shiraz, Iran; 3 Department of Electrical and Computer Engineering, Isfahan University of Technology, Isfahan, 84156-83111 Iran; 4 Department of Computer Engineering, Faculty of Engineering, University of Birjand, Birjand, Iran; 5 Department of Computer Engineering, Amol Institute of Higher Education, Amol, Iran; 6 FPGA Laboratory, Faculty of Electrical Engineering, K. N. Toosi University of Technology, Tehran, Islamic Republic of Iran; 7 Delbeat Inc., Berkeley, CA USA; 8 Dibrugarh University, Assam, 786004 India; 9 Omid Hospital, Iran University of Medical Sciences, Tehran, Iran; 10 Department of Medical Engineering, Mashhad Branch, Islamic Azad University, Mashhad, Iran; 11 Department of Cardiology, National Heart Centre Singapore, Singapore, Singapore; 12 Department of Electronics and Computer Engineering, Ngee Ann Polytechnic, Singapore, Singapore; 13 Department of Biomedical Engineering, School of Science and Technology, Singapore University of Social Sciences, Singapore, Singapore; 14 Department of Bioinformatics and Medical Engineering, Asia University, Taichung City, Taiwan; 15 School of Exercise and Nutrition Sciences, Institute for Physical Activity and Nutrition, Deakin University, Geelong, VIC 3220 Australia; 16 Cardiovascular Division, The George Institute for Global Health, Newtown, Australia; 17 Sydney Medical School, University of Sydney, Camperdown, Australia

**Keywords:** Data mining, Image processing, Machine learning, Coronary artery disease and stable angina

## Abstract

Coronary artery disease (CAD) is a prevalent disease with high morbidity and mortality rates. Invasive coronary angiography is the reference standard for diagnosing CAD but is costly and associated with risks. Noninvasive imaging like cardiac magnetic resonance (CMR) facilitates CAD assessment and can serve as a gatekeeper to downstream invasive testing. Machine learning methods are increasingly applied for automated interpretation of imaging and other clinical results for medical diagnosis. In this study, we proposed a novel CAD detection method based on CMR images by utilizing the feature extraction ability of deep neural networks and combining the features with the aid of a random forest for the very first time. It is necessary to convert image data to numeric features so that they can be used in the nodes of the decision trees. To this end, the predictions of multiple stand-alone convolutional neural networks (CNNs) were considered as input features for the decision trees. The capability of CNNs in representing image data renders our method a generic classification approach applicable to any image dataset. We named our method RF-CNN-F, which stands for Random Forest with CNN Features. We conducted experiments on a large CMR dataset that we have collected and made publicly accessible. Our method achieved excellent accuracy (99.18%) using Adam optimizer compared to a stand-alone CNN trained using fivefold cross validation (93.92%) tested on the same dataset.

## Introduction

Coronary artery disease (CAD) is a prevalent condition that affects a growing number of people worldwide. In the United States, about 18.2 million Americans ≤ 20 years of age have coronary heart disease. One American will suffer a heart attack every 40 s, and more than 350,000 will die from coronary heart disease every year^1^. Early diagnosis of CAD facilities intensification of guideline-directed medical therapy and, if indicated, coronary intervention, to avert major adverse cardiac events and improve clinical outcomes.

Clinicians cannot rely on patient’s symptoms alone to diagnose CAD as they are neither sensitive nor specific. Heart imaging can help the physicians to detect CAD earlier and treat patients more effectively. The reference standard for diagnosis of CAD is invasive coronary angiography. CAD is diagnosed with more than 50% stenosis of the left main, left anterior descending, circumflex or right coronary artery. However, invasive coronary angiography is expensive and carries potential risks. Cardiac imaging is cheaper, safer, and can help physicians confidently diagnose CAD noninvasively. Thereby imaging alone can serve as a gatekeeper to downstream invasive coronary angiography and definitive revascularization therapy. Stress electrocardiography, echocardiography, nuclear myocardial perfusion scans, and coronary computed tomographic angiography (CCTA) are the mainstays of noninvasive imaging tests for CAD. However, CCTA suffers from more than 30% false positive rate^2^. To reduce the number of misdiagnosed cases, HeartFlow Inc. developed a noninvasive image and physics-based technology to reduce the number of unnecessary invasive coronary angiography. However, the HeartFlow Inc. approach needs a few days for analysis, and it still has more than 20% error.

Not being limited by acoustic window access (as in echocardiography) or ionizing radiation concerns (as in nuclear imaging and CCTA), cardiac magnetic resonance (CMR) has emerged as a viable alternative for noninvasive assessment of CAD^3^. It provides precise measurements of heart chamber structure and function, myocardial perfusion and infarct extent, as well as parametric quantitation of myocardial tissue characteristics. These readouts facilitate comprehensive CAD diagnosis, disease surveillance, and monitoring of therapeutic response^4,5^.

The manual interpretation of diagnostic imaging tests requires time and expertise. Moreover, data-mining and machine learning methods are exploited to automate medical diagnoses to reduce analysis time and potentially improve accuracy. CMR images may be 2D or 3D. The sensitivity and specificity of 3D CMR of the coronary arteries is inadequate due to excessive artefacts. This explains why 3D CMR coronary angiography for CAD diagnosis has not become a clinical routine. On the contrary, CMR 2D cine images are commonly used to diagnose CAD based on indirect evidence of myocardial ischemia or infarct, such as segmental regional wall motion abnormalities and myocardial thinning in typical coronary arterial territories. Regarding artificial intelligence-enabled diagnosis, deep models can conglomerate pixel-level features in an image to a degree that is not possible with the human eye. The excellent results of our model for detection of CAD attests to this postulation. Based on the description above, in this paper, we proposed the use of deep learning combined with a random forest classifier to analyse CMR 2D images for CAD diagnosis. The main contributions of this paper are as follows:

- We have collected and made publicly available a dataset comprising multiparametric CMR images that can be used to train, test, and validate automated CAD detection methods.
- We have developed a novel ensemble approach for diagnosing CAD on CMR images. The method deployed convolutional neural networks (CNNs) to convert high-dimension CMR images to low-dimension numeric features, which were then used to build the nodes of random forest decision trees. Without CNNs, each pixel of the CMR image would have been considered one feature, which would render the random forest intractable due to an inordinately high number of features.
- CNNs are designed to extract the essential features of any image dataset automatically. Therefore, using CNNs predictions as features enhances the generalizability of the proposed method.
- By using an ensemble of decision trees, our method was able to achieve high classification accuracy.

In the remainder of this paper, related works are reviewed (“Related work” Section), background is discussed (“Background ” Section), the dataset is introduced (“Dataset description” Section), the proposed method is described (“Proposed method” Section), and experimental results are presented (“Experimental results” Section). Additionally, discussion and conclusion are presented in “Discussion and Conclusion” Sections, respectively.

## Related work

To the best of our knowledge, our work is the first that uses CMR data as input to ensemble of deep neural networks in order to diagnose CAD. Therefore, in this section, existing CAD diagnosis methods that use other input data such as electrocardiography (ECG), phonocardiography (PCG), etc. are reviewed. This way we can compare our method based on CMR with existing ones based on other data types. We also review some of the ensemble-based methods since our method uses an ensemble of decision trees for CAD diagnosis.

### ECG

Butun et al.^6^ reported a technique for automated diagnosis of CAD from ECG signals based on capsule networks (CapsNet). They applied the 1D version of CapsNet for automated detection of CAD ("1D-CADCapsNet" model) on two- and five-second ECG segments, and attained optimal diagnostic accuracy of 98.62% for five-second ECG signals. Acharya et al.^7^ proposed a CNN comprising four convolutional, three fully-connected, and four max-pooling layers that achieved 94.95% and 95.11% accuracy rates for discriminating between abnormal (from patients with CAD and myocardial infarct) and normal ECGs on two- and five-second ECG segments, respectively. Khan et al.^8^ presented a signal processing framework to diagnose CAD on raw ECG signals of 9–12 min durations. The ECG signals were pre-processed and segmented using empirical mode decomposition by selecting intrinsic mode function 2–5. The features for optimal classification of data included marginal factor, impulse factor, shape-factor, kurtosis, etc. The pre-processed signals were fed to the support vector machine classifier, and achieved an accuracy of 95.5%.

### PCG

The PCG records heart sounds and murmurs that may become altered in CAD, but these changes can be subtle and imperceptible to the human ear. To overcome the low signal-to-noise ratio of PCG signals due to environmental noise, Pathak et al.^9^ developed a PCG-based CAD detection method. They recorded PCG signals from four auscultation sites on the left anterior chest using a multichannel data acquisition system. Evaluated in the presence of white noise, vehicle and babble, the system was effective in collecting essential information from both diastolic and systolic phases of the cardiac cycle. Li et al.^10^ introduced a new feature fusion approach that fed Mel-frequency cepstral coefficients to a CNN to output valuable features that were in turn fused and provided to a multilayer perceptron for classification. In subsequent work, Li et al.^11^ demonstrated the efficacy of a new dual-input neural network that analyzed simultaneously assembled PCG and ECG signals using combined deep learning and feature extraction to extract useful underlying information in the signals.

### Blood assays

Dyslipidemia is a pathogenetic factor that contributes to the development of CAD. Guo et al.^12^ investigated whether the atherogenic index of plasma (AIP) is an independent predictor of CAD risk in postmenopausal women. Compared with controls, triglyceride (TG) was higher, high-density lipoprotein cholesterol (HDL-C) levels lower, and non-traditional lipid profile values (AIP, total cholesterol/ HDL-C ratio) more elevated in CAD subjects. Circular RNAs (circRNAs) have emerged as a potential biomarker for CAD. Liang et al.^13^ elucidated the role of circZNF609 in atherosclerosis. They demonstrated on logistic regression an independent inverse relationship between circZNF609 expression (quantitated in peripheral blood leukocytes using real-time polymerase chain reaction) and CAD risk among 209 controls and 330 CAD patients.

### Clinical features and CCTA

Al’Aref et al.^14^ used a machine learning method to predict obstructive CCTA using clinical factors and the coronary artery calcium score. They employed the boosted ensemble algorithm XGBoost with tenfold cross-validation. Age, gender, and coronary artery calcium score were found to be the most prominent features. Baskaran et al.^15^ validated a machine learning model to predict obstructive CAD and revascularization on the Coronary Computed Tomographic Angiography for Selective Cardiac Catheterization (CONSERVE) study dataset^16^, which outperformed the CAD consortium clinical score. Imaging variables were most correlated with revascularization, and the performance did not differ whether the imaging parameters used were derived from invasive coronary angiography or CCTA.

### Ensemble methods

Since our method is based on ensemble of decision trees, we review some of the existing literature on CAD diagnosis which use ensemble of classifiers. In^17^_bookmark44, three base classifiers namely K-Nearest-Neighbor, random forest and SVM are combined to form an ensemble method for CAD diagnosis. The final decision is made based on ensemble voting techniques. The experiments were carried out on Z-Alizadeh Sani dataset^18^. Another method based on rotation forest algorithm has used neural networks as the base classifiers^19^_bookmark46. The method has been evaluated on Cleveland dataset. Kausar et al.^20^ combined the advantages of supervised and unsupervised methods by utilizing K-means and SVM. They performed dimensionality reduction via principle component analysis and tested their method on Cleveland dataset.

Abdar et al.^21^ used nu-SVC as the base algorithm to present NE-nu-SVC method for CAD diagnosis. To gain better results, the authors balanced the studied datasets (Cleveland and Z-Alizadeh Sani) and performed feature selection. Finally, Hedeshi et al.^22^ took a PSO-based approach to extract set of rules for CAD diagnosis. The extracted rules were reported to have good Interpretability.

## Background

Considering that the proposed method is based on random forest and decision tree, these methods are briefly reviewed in this section.

### Decision tree

A decision tree can be considered a series of Yes/No questions asked to make predictions about data. Decision trees are interpretable models since they carry out classification much as humans do. A series of questions are answered to arrive at a conclusion. The decision tree nodes are created that minimizes Gini impurity measures. For each node, the Gini impurity measure is the probability that a randomly drawn sample from the node is misclassified. As nodes of the tree are created, Gini impurity is reduced. Any node with zero Gini impurity is considered a leaf node and is not expanded anymore. For a classification problem with C classes, the Gini impurity of a node n is computed as.

$$I_{G} \left( n \right) = 1 - \mathop \sum \limits_{i = 1}^{c} p\left( i \right)^{2} ,{ }$$

where *p*(*i*) is the probability of picking a sample with class i in node n.

**Figure 1:**
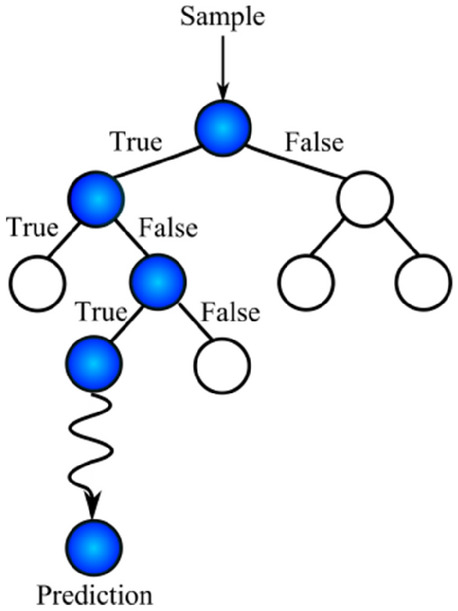
Demonstration of a typical decision trees.

A typical decision tree is presented in Fig. 1. At each node, a specific condition is checked with the input sample. Depending on the situation being True or False, one of the two offsprings of the node is chosen. The routine continues until a leaf node is reached or some termination condition is met^18^.

### Random forest

**Figure 2:**
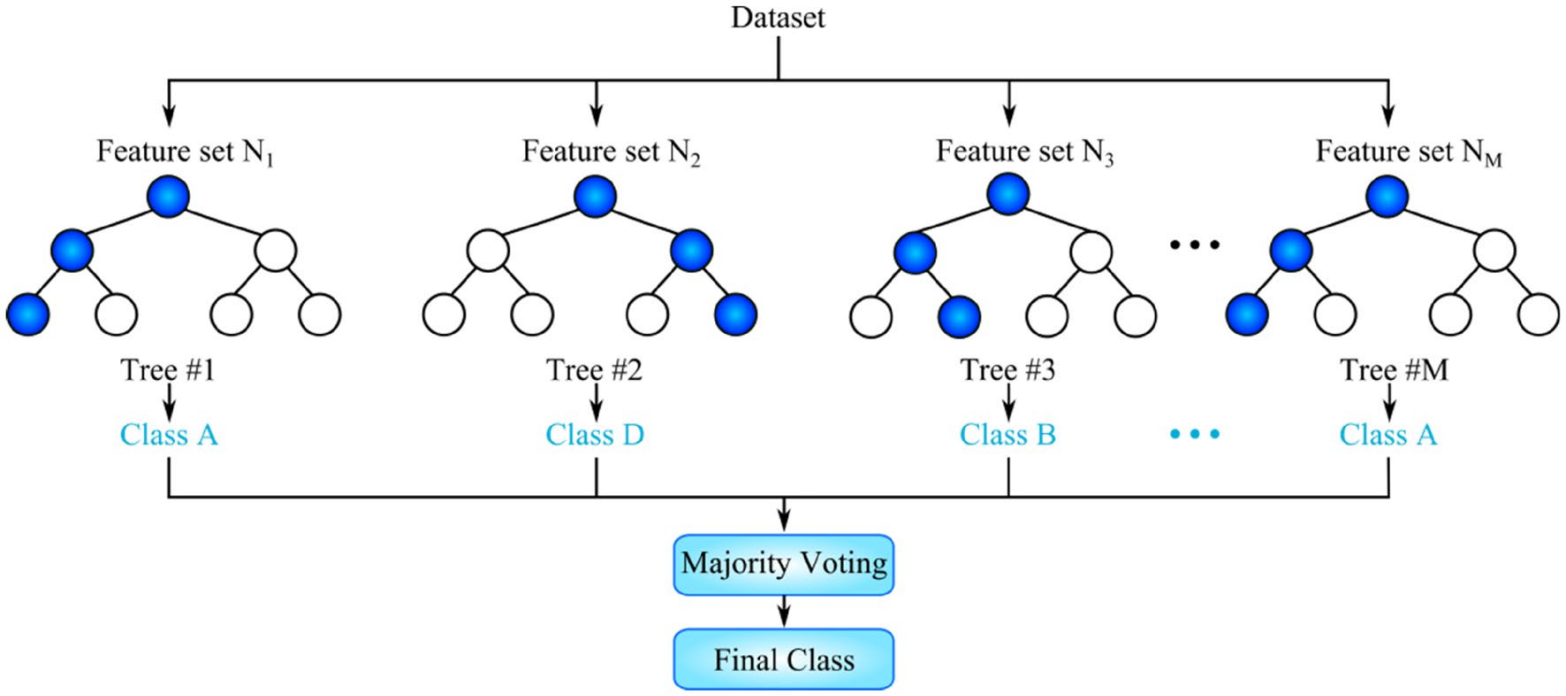
Schematic of a random forest with M number of decision trees. The final classification is determined by majority voting of the classification results of individual decision trees.

Random forest is a model that contains multiple decision trees. To build each of the trees, a random subset of the training data is used. The samples that form the subset are drawn with replacement from the training data. Therefore, some samples may be used multiple times in a single tree. Moreover, to split tree nodes, a random subset of features is considered. During the testing phase, each decision tree of the forest makes a prediction about the given test sample. The final prediction result is then decided by majority voting (Fig. 2)^23^. Using multiple trees, the random forest achieves good prediction accuracy by avoiding the overfitting that a single decision tree may be prone to. Each of the decision trees is built using a random subset of data features. For classification problems, the subset cardinality is usually set as $\sqrt{\#features}$. Assuming the dataset has 36 features, six features will typically be considered to split each tree node. This is by no means immutable. For instance, it is common to deploy all features for node splitting in regression problems.

## Dataset description

**Figure 3:**
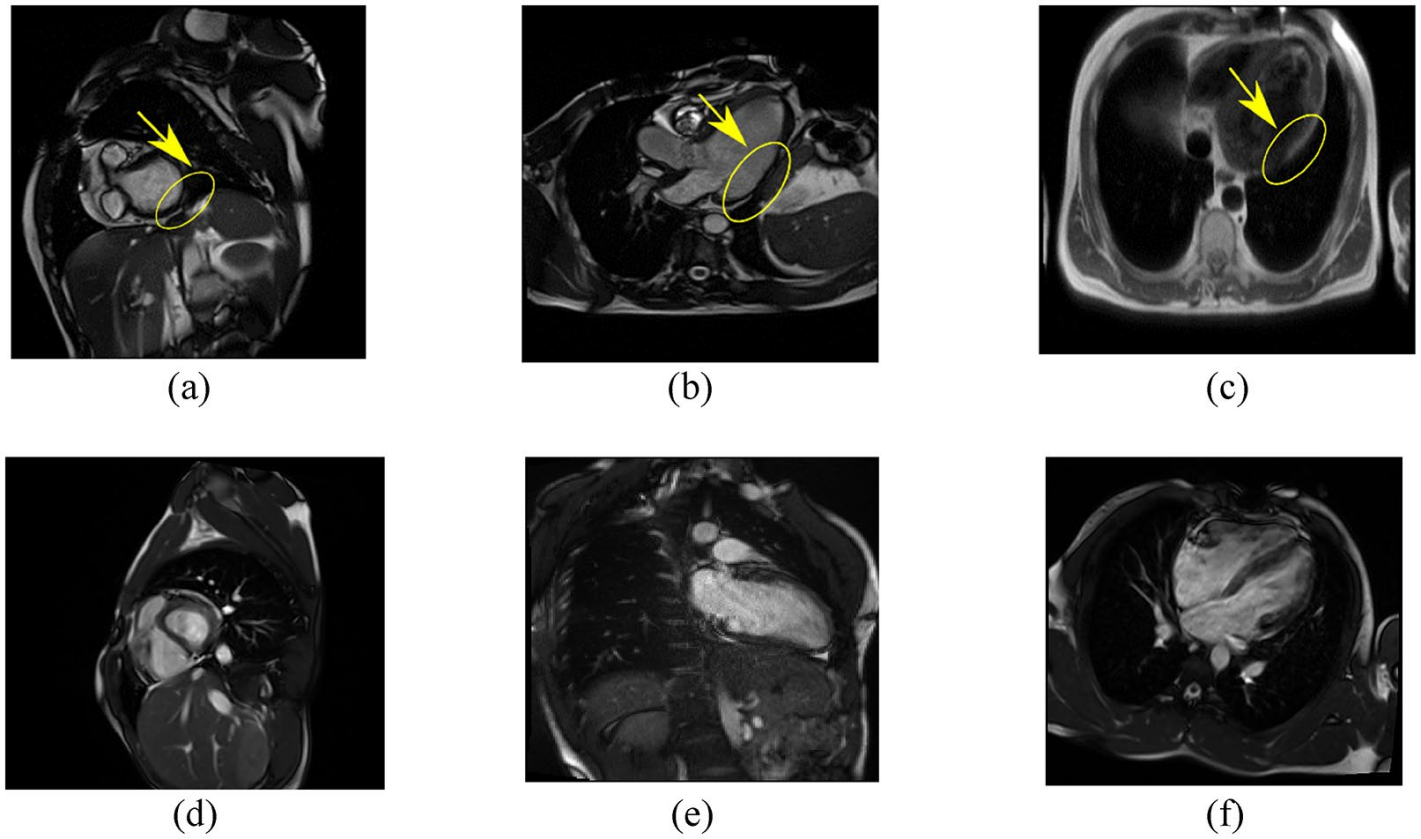
Example cardiac magnetic resonance images from coronary artery disease patients (**a**–**c**) and healthy subjects (**d**–**f**). c is a black-blood spinecho image; the rest are single-phase images of steady-state free precession CINE images. The lesions indicating CAD have been marked with yellow color in parts (**a–c**).

**Figure 4:**
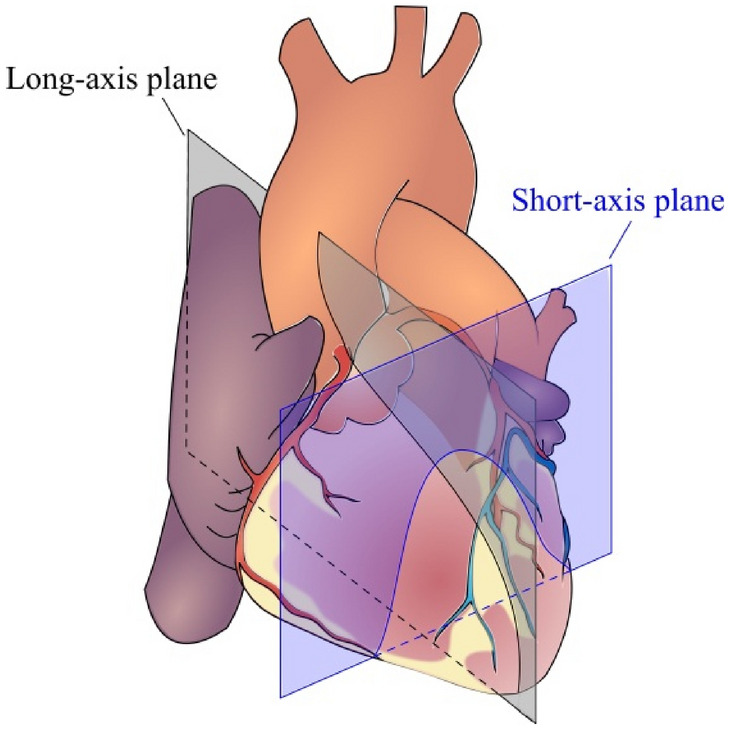
Illustration of long and short axes planes used during collecting CMR images of our dataset.

The dataset contains 63,648 multiparametric CMR images. The number of images with CAD disease is 26,104 and the number of images representing healthy patients is 37,544 (see examples of both in Fig. 3). The dataset is publicly accessible^24^. In the patient group, CAD was confirmed on invasive coronary angiography, the diagnostic gold standard. To collect the CMR images, four types of sequences namely LGE, Perfusion, T2 weighted, and SSFP have been used. In each of these sequences, long and short axes planes of the heart have been considered (Fig. 4). Long axis consists of two, three, and four chamber views. For each of these views, one slice from 2 different angles has been captured. Short axis consists of 10 slices from base to apex of the heart. Again, each of the 10 slices has been captured from 2 different angles. Based on the description above, for each patient 13 slices have been collected in four different sequence types. Therefore, 13 × 4 CMR images have been collected per patient. Dividing the total number of CMR images (63,648) in the dataset by 13 × 4 reveals that 1224 patients (722 healthy and 502 CAD) have participated in the collection of this dataset.

To be able to use the collected dataset in research studies regarding diagnostic and therapeutic purposes, institutional approval was granted. Approval was granted on the grounds of existing datasets. Before collecting data, patients were informed about the data collection process and their consent were obtained. Execution of all methods was compliant with relevant guidelines and regulations. To use data, ethical approval was obtained from Tehran Omid hospital.

### CMR imaging protocols

From September 2018 to September 2019, the study prospectively recruited subjects who attended the CMR department of Omid Hospital, Tehran, Iran. The institutional ethics committee of Tehran Omid hospital had approved the study. All participants gave written informed consent. CMR examination was performed on a 1.5-T system (Magnetom Aera Siemens, Erlangen, Germany) using dedicated body coils. In each subject, segmented true fast imaging with steady-state precession (TrueFISP) cine images as well as high-resolution phase-sensitive inversion-recovery (PSIR) early (EGE, immediately after bolus infusion of gadoterate meglumine contrast at 0.1 mmol/kg body weight) and late gadolinium enhancement (LGE, 15 min after contrast administration) images were acquired in the standard long- (LAX) and short-axis (SAX) views. Parametric maps of left ventricular myocardial T2, native T1, and post-contrast T1 (10–30 min after contrast administration) were acquired in the basal, mid and apical SAX views. Table 1 summarizes the details of CMR sequences and typical parameters.

**Table 1:** Details of CMR sequences and typical parameters.

Parameters							
Sequence	TE (ms)	TR (ms)	Segment length	Slice thickness (mm)	Concentration/number of slices	NEX	Breath hold time (s)
Cine, segmented TrueFISP; LAX/	1.15	33.60	15	7	3	1	8
Cine, segmented TrueFISP; SAX	1.11	31.92	15	7	15	1	8
Dynamic TrueFISP (during contrast infusion); 3 SAX	2.48	412.78	74	8	Slice No: 3	1	Free breathing
EGE, high-resolution PSIR; LAX and SAX	3.16	666	Non-cine	8	1	1	7
LGE, high-resolution PSIR; SAX and LAX	3.16	666	Non-cine	8	1	1	7
Myocardial T2; SAX	1.06	193.27	56	8	3	1	9
Native myocardial T1; SAX	1.12	280.56	72	8	3	1	9–10
Post-contrast myocardial T1; SAX	1.12	360.56	72	8	3	1	9–10

*NEX* Number of excitations, *TE* Echo time, *TR* Repetition time.

## Proposed method

The motivation behind the proposed method and its detailed description are explained in this section.

### Motivation

- Each pixel of an image forms a meaningful pattern connecting neighbouring pixels. The conversion of pixels to feature vectors would delink all interpixel relationships leading to violation of pixel locality and severe degradation of classification performance.
- To feed image data to a decision tree, we would be forced to treat each pixel as one feature. This would increase the number of features inordinately, thereby incurring the curse of dimensionality.

As such, it became apparent that we had to use a different approach than the pixel-based method to convert the whole image into numeric features that can be fed efficiently to the decision trees. To this end, we trained CNNs on image data and used the CNN outputs as input features for the decision trees of the random forest. CNNs can extract essential features from whole input images^26^ for accurate prediction, thereby avoiding the curse of dimensionality. Moreover, with CNNs, the locality of the pixels is preserved^27^.

It is known that training the same CNN multiple times will lead to a different parameter set. The reason is two-fold. First, each run uses a different random seed leading to different initialization of CNN parameters. Second, CNNs are trained on mini-batch of samples drawn randomly from the training set. The stochastic nature of mini-batch sampling affects the overall training process. Therefore, one training run might result in a better parameter set than the other. To reduce the unwanted effects of stochastic training, we train multiple CNNs employing K-fold cross validation and use their prediction during random forest creation leading to better overall performance. Given that we used stratified K-fold cross validation, the percentage of healthy and CAD samples within each fold was preserved. Recall that in our dataset, the total numbers of healthy and CAD patients were 722 and 502, respectively. Therefore, in each fold, the numbers of healthy and CAD patients were approximately 144 and 100, respectively.

### Proposed method description

**Figure 5:**
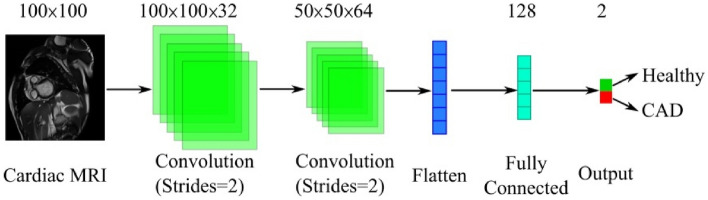
The architecture of convolutional neural networks used in the proposed method. CAD, coronary artery disease; CMR, magnetic resonance imaging.

The proposed method used a combination of random forest and CNNs. Using CNNs, 2D CMR images were converted into vectors of real values automatically. These vectors were fed to the random forest of decision trees instead of direct pixel-by-pixel feeding of CMR images as the latter would otherwise have led to violation of pixel locality (i.e., loss of inter-pixel spatial relationships) and incurred the curse of dimensionality. The CNNs could be thought of as the “feature generation” step that preceded the “classification” step comprising the random forest of decision trees. The structure of each of the CNNs is depicted in Fig. 5. At the pre-processing phase, input data were resized to $100\times 100$ and normalized between 0 and 1. Next, the dataset *D* was divided into five parts for fivefold cross validation. The training was repeated five times for k = 1,…, 5. In each iteration k, the training set ${f}_{k}\subset D$ consisted of data from four out of the five folds. The remaining fold was used for testing. To train the CNNs $\{{C}_{i}, i=1, \dots , N\}$, each training set ${f}_{k}$ was divided to n subsets $\{{f}_{k1}, \dots , {f}_{kn}|{f}_{kl}\cap {f}_{km}=\varnothing , l,m\in \{1,\dots ,n\}\wedge l\ne m\}$. The number of CNNs (n) is the hyperparameter of the proposed method which was set to 10 in our experiments.

To ensure that the CNNs would have different sets of parameters, each CNN ${C}_{i}, i=1,\dots ,n$ was trained on subset ${f}_{k}-{f}_{ki}$ and validated on subset ${f}_{ki}$. The trained CNNs were used to create the random forest decision trees. Each node of a decision tree represented one randomly selected CNN-generated numerical feature of a randomly selected CMR image sample of the training set. Computationally, one of $|{f}_{k}|$ rows and one of n columns were randomly selected from the $[|{f}_{k}|\times n]$ matrix of outputs obtained by feeding $\left|{f}_{k}\right|$ CMR images to n CNNs for each fold k of K-fold cross validation.

**Figure 6:**
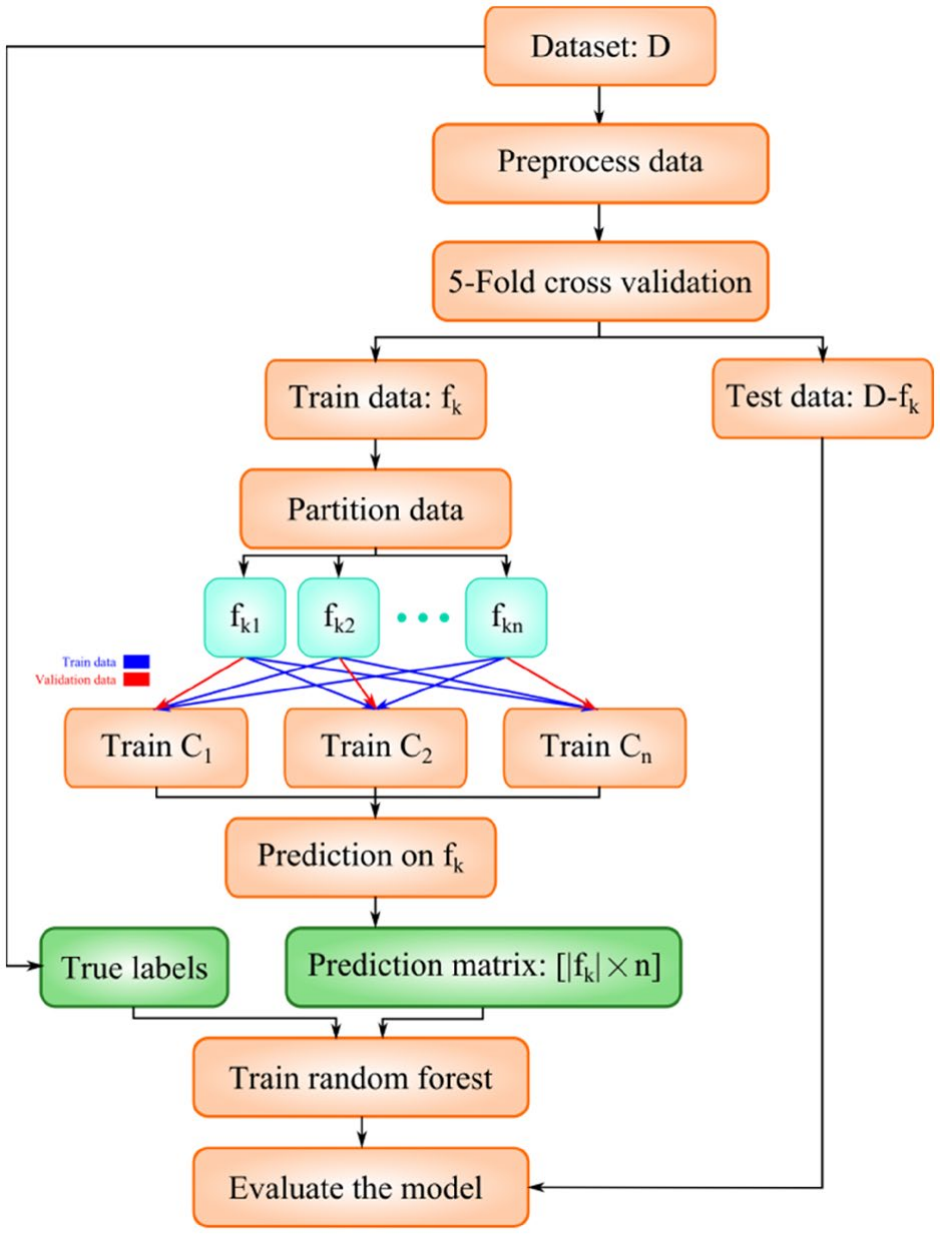
Steps in the proposed method.

Finally, the test set $D-{f}_{k}$ was used to evaluate the trained model and the final results were presented. To this end, each test sample was fed to the CNNs and their outputs were used as input to the random forest. Based on the CNNs outputs, the decision trees of the random forest determined whether the test sample had CAD disease or not. The final prediction was presented using majority voting between the decision trees predictions. High-level steps of the proposed method are presented in Fig. 6. In contrast to conventional studies where validation is only performed after obtaining the final output of the classifier, training and validation were conducted for each CNN during the “feature generation step” as well as for the random forest of decision trees based on the aggregated results of the former during the “classification” stage (see Fig. 6).

**Figure 7:**
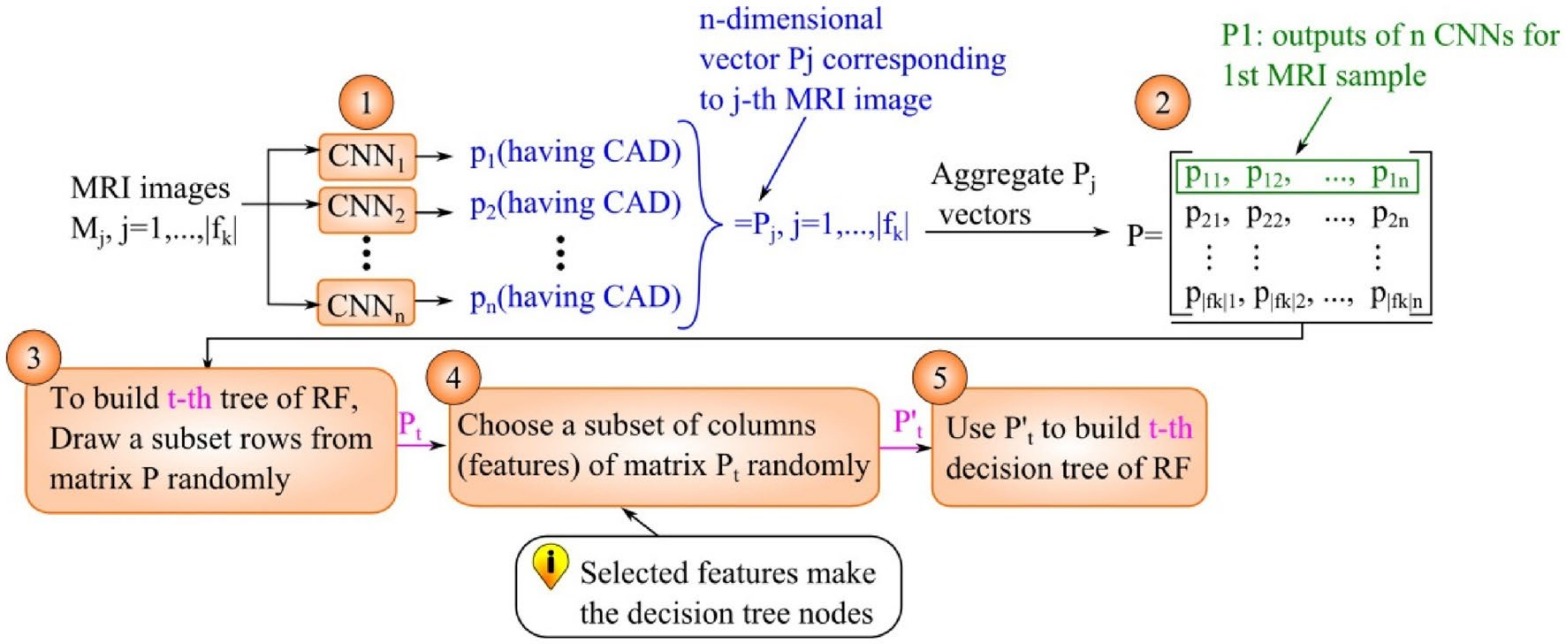
The graphical representation of operations in lines 8–19 of Algorithm 1.


The pseudo-code of the proposed method is presented in Algorithm 1. In lines 1–2, the training data are pre-processed. Next, the K-fold cross validation loop is begun. Training data for k-th iteration (${f}_{k}$) is divided to n subsets. The subsets are used for training of n CNNs in lines 6–7. The trained CNNs are used to compute matrix P in lines (8–11). Based on matrix P, decision trees of RF are built one by one in lines (13–19). For better clarification, the operations performed in lines (8–19) of Algorithm 1 are depicted in Fig. 7.

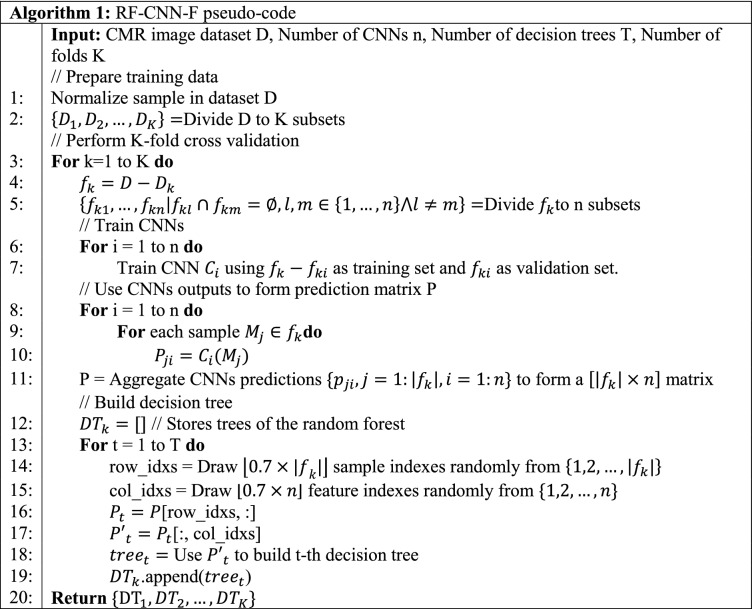


## Experimental results

All the experiments were implemented in Python using the Keras library. The models were trained using GeForce GTX 950 GPU and 16 GB of RAM. Further, we compared our method with a CNN (as a single classifier) to investigate the effect of using an ensemble of classifiers versus just one. As mentioned in “Random forest” Section, n = 10 CNNs were used to convert the image dataset into n numeric features that were required to build the random forest decision trees nodes. The hyperparameters of the CNNs are summarized in Table 2. We used 15 decision trees in the random forest. The experiments were conducted in two phases. First, CNNs were trained for 10 epochs following the procedures explained in “Random forest” Section _bookmark9 and Fig. 6. Next, the trained CNNs were used to generate the numeric features that were required for the creation of the decision trees. We compared our method with a single CNN that was trained using fivefold cross validation. To make the comparison fair, the training time assigned to the single CNN was equal to the total training time of our approach. In other words, the CNNs used in our approach were trained only for 10 epochs but the comparator single CNN model was trained for as many epochs as possible until reaching the total training time of our method (464.75 s). The effect of using different optimizers has also been inspected. To this end, the experiments have been performed for three optimizers namely Adagrad^28^, RMSProp^29^, and Adam^30^. The performance of our method and the stand-alone CNN are compared in Table 3, which shows the superiority of our method. As can be seen, regardless of the optimizer type, our method has outperformed the stand-alone CNN which shows that our method is not sensitive to the choice of the optimizer. Moreover, among the evaluated optimizers, Adam has yielded the best performance. In Table 3, we did not include loss function for our method since it is a hybrid approach.

**Table 2:** Hyperparameters used to train the CNNs used in our experiments.

Hyperparameter	Value
Input dimension	100 × 100
Number of convolution layers	2
Number of fully connected layers	1
Number of filters for each convolution layer	32, 64
Size of convolutional kernels	3 × 3
Strides size	2
Activation function for hidden layers	ReLU
Loss function	Hinge
*L*_2_ regularization coefficient	0.001
Number of neurons of fully connected layers	128
Batch size	256

**Table 3:** Performance comparison between a stand-alone classifier (CNN) and the proposed method.

Optimizer	Methods	Accuracy (%)	PPV (%)	Recall (%)	Specificity (%)	F1-score (%)	AUC	Loss	Total training time (s)
Adagrad	CNN	92.45	93.91	87.02	94.91	90.89	0.91	0.52	464.75
	Proposed method	98.78	100	98.16	99.42	99.00	0.99	–	464.75
RMSProp	CNN	93.48	94.56	89.99	95.13	91.03	0.93	0.48	471.22
	Proposed method	98.99	100	98.65	99.49	99.50	0.99	–	471.22
Adam	CNN	93.92	95.01	90.09	95.89	92.22	0.95	0.41	476.85
	Proposed method	99.18	100	98.88	99.66	99.70	0.99	–	476.85

We have compared the performance of our method using CMR data with existing ones using other data types in Table 4. To ease the comparison between our method and existing ones, the last row of Table 3 has been repeated as the last row of Table 4. Clearly, our method has outperformed the rival ones in Table 4 which suggests that CMR can be used as a reliable data source for CAD diagnosis.

**Table 4:** Overview of related works based on various input types.

Refs.	Method	Input data type	Detection task	Performance %
^31^	Time–frequency analysis of PCG signal using chirplet transform	PCG	Valve disease diagnosis	Accuracy 98.33
^32^	Recurrent neural network with long short-term memory	CCTA	Calcified plaque detection	Accuracy 90.3 Sensitivity 92.1 Specificity 88.9
^33^	CNN	ECG	Diagnosis of different cardiovascular diseases	Accuracy 95
^34^	Optimal time–frequency concentrated biorthogonal wavelet-based features	ECG	CAD diagnosis	Accuracy 98.53
^35^	Binomial rendition of the bivariate mixed-effects regression model	CCTA, ECG	CAD diagnosis	Sensitivity 99 Specificity 88
^36^	Discrete wavelet transform, multivariate multi-scale entropy,	ECG	CAD diagnosis	Accuracy 98.67
^37^	Sequential minimal optimization, Naive Bayes, and ensemble algorithm	ECG	CAD diagnosis	Accuracy 88.5
^38^	Computing complex ventricular excitation index	Magneto-cardiography	CAD diagnosis	Sensitivity 91 Specificity 84
^39^	Extracted time- and frequency-domain features from PCG signal as inputs to neural network classifier	PCG	CAD diagnosis	Accuracy 82.57 Sensitivity 85.61 Specificity 79.55
^40^	Multimodal feature fusion and hybrid feature selection, SVM classifier	ECG, PCG	CAD diagnosis	Accuracy 96.67 Sensitivity 96.67 Specificity 96.67 F1-measure 96.64
^41^	Multimodal feature fusion, SVM classifier	PCG, PPG	CAD diagnosis	Sensitivity 80 Specificity 93
^42^	Combined feature set related to heart rate variability and shape of PPG waveform, SVM classifier Two sets of features extracted from	PPG	CAD diagnosis	Sensitivity 73 Specificity 87
^43^	Two sets of features extracted from PPG and PCG, SVM classifier	PCG, PPG	CAD diagnosis	Sensitivity 92 Specificity 90
^44^	Novel feature representation using synchrosqueezing transform, CAD diagnosis based on entropy of PCG, SVM classifier	PCG	CAD diagnosis	Accuracy 83.48
^45^	Hybrid neural network-genetic algorithm	Echo	CAD diagnosis	Accuracy 93.85 Sensitivity 97 Specificity 92
^46^	Sequential minimal optimization Naive Bayes, C4.5 and AdaBoost	Laboratory data, echo	CAD diagnosis	Accuracy 82%
^19^	Rotation forest with neural networks as base classifiers	Cleveland	CAD diagnosis	Accuracy 91.20 AUC 91.50 Sensitivity 95.60 Specificity 86.70
^21^	Nested ensemble nu-Support Vector Classification	Z-Alizadeh Sani	CAD diagnosis	Accuracy 94.66 Precision 94.70 Sensitivity 94.70
^22^	Ensemble PSO-based fuzzy rule extraction	Cleveland	CAD diagnosis	Accuracy 92.59 Specificity 94.37 Sensitivity 90.51
Proposed method	Random forest, CNNs as feature extractors, Adam optimizer	CMR	CAD diagnosis	Accuracy 99.18 Sensitivity 98.88 Specificity 99.66 AUC 99

*ECG* Electrocardiograph, *Echo* Echocardiography, *PCG* Phonocardiograph, *PPG* Photoplethysmography, *SVM* Support vector machine.

## Discussion

Random forests have proved to be robust and accurate in challenging classification problems. As they are designed to work with numeric features, feeding image data pixel by pixel directly to random forests causes them to incur the curse of dimensionality. Even for a small image 28 × 28, the random forest will have to deal with 28^2^=784 features, which renders the learning process prohibitively demanding. To this end, we relied on the automatic feature extraction property of CNNs to convert images to useful numeric values. However, as CNNs get more complex, the training time increases. As the random forest comprises an ensemble of decision trees, we can train multiple lightweight CNNs (with moderate accuracy) in a shorter time and still achieve good performance by harnessing an ensemble of decision trees that use CNNs predictions as input features. Despite being slow to train, the CNNs could perform quite fast during the testing phase. Once trained, the model can deliver real-time performance.

The advantages of the proposed method are as follows:

- Using random forest for image data classification reinforces the reliability of the prediction since an ensemble of decision trees is used.
- Since CNNs perform automatic feature extraction, we do not need to design hand-crafted features. This enhances the generalizability of our method.
- The proposed method is applicable to any image-based classification problem as CNNs can learn any image dataset. Therefore, we can train a reasonable number of CNNs on the dataset and use their predictions as features in random forest decision trees.

The disadvantages of our method are discussed below:

- Complex problems may demand more features to achieve acceptable accuracy. Considering that we rely on CNNs to map images to useful numeric features, the training time of our method will increase commensurately with the increased number of features.
- The performance of the random forest is affected by the number of features used in the decision trees. In our method, each feature corresponds to the prediction of a trained CNN. Currently, the number of CNNs (features) is treated as a hyperparameter that is set by trial and error. This introduces a degree of arbitrariness to our method. However, it is possible to use random forests to choose features that play more significant roles in classification performance. Conceivably, we can start by training several CNNs and then use the random forest to screen for the CNNs in which the predictions are more critical in classification performance. The CNNs that are less important can then be discarded. Such an approach constitutes an interesting direction for future work.
- The dataset samples used in the experiments have been obtained in a single institution. It is desirable to validate the model on other datasets.

## Conclusion

In this paper, a novel classification method was presented for diagnosing CAD patients based on ensemble of CNNs and random forest. The method harnesses the classification power of multiple decision trees of the random forest. We trained multiple CNNs on the image dataset, and the trained CNNs predictions were used as features during the decision tree building process. Our method was able to achieve high accuracy using lightweight CNNs to derive features. We reported the classification results on our CMR image dataset, which has also been released for public use. By exploiting the representation capability of CNNs, the proposed method has enabled the use of random forest for the classification of any image-based dataset.

## Data Availability

The datasets analysed during the current study are available in the CAD Cardiac MRI Dataset repository, https://www.kaggle.com/danialsharifrazi/cad-cardiac-mri-dataset.
